# Role of Muramyl Dipeptide in Lipopolysaccharide-Mediated Biological Activity and Osteoclast Activity

**DOI:** 10.1155/2018/8047610

**Published:** 2018-02-14

**Authors:** Hideki Kitaura, Masahiko Ishida, Keisuke Kimura, Haruki Sugisawa, Akiko Kishikawa, Kazuhiro Shima, Saika Ogawa, Jiawei Qi, Wei-Ren Shen

**Affiliations:** Division of Orthodontics and Dentofacial Orthopedics, Department of Translational Medicine, Tohoku University Graduate School of Dentistry, 4-1 Seiryo-machi, Aoba-ku, Sendai 980-8575, Japan

## Abstract

Lipopolysaccharide (LPS) is an endotoxin and bacterial cell wall component that is capable of inducing inflammation and immunological activity. Muramyl dipeptide (MDP), the minimal essential structural unit responsible for the immunological activity of peptidoglycans, is another inflammation-inducing molecule that is ubiquitously expressed by bacteria. Several studies have shown that inflammation-related biological activities were synergistically induced by interactions between LPS and MDP. MDP synergistically enhances production of proinflammatory cytokines that are induced by LPS exposure. Injection of MDP induces lethal shock in mice challenged with LPS. LPS also induces osteoclast formation and pathological bone resorption; MDP enhances LPS induction of both processes. Furthermore, MDP enhances the LPS-induced receptor activator of NF-*κ*B ligand (RANKL) expression and toll-like receptor 4 (TLR4) expression both *in vivo* and *in vitro*. Additionally, MDP enhances LPS-induced mitogen-activated protein kinase (MAPK) signaling in stromal cells. Taken together, these findings suggest that MDP plays an important role in LPS-induced biological activities. This review discusses the role of MDP in LPS-mediated biological activities, primarily in relation to osteoclastogenesis.

## 1. Introduction

Lipopolysaccharide (LPS) is a major component of the cell wall of Gram-negative bacteria and is an inflammation-inducing endotoxin [1–6]. Exposure to LPS can induce proinflammatory cytokines, such as tumor necrosis factor- (TNF-) *α* and interleukin- (IL-) 1, from macrophages or other cells in the affected area [7, 8]. Peptidoglycan (PGN) is a major component of the cell membranes of both Gram-negative and Gram-positive bacteria. Muramyl dipeptide (MDP) is the minimal essential structural unit of PGN responsible for its immunological activity. Coinjection of MDP and LPS into mice enhances production of proinflammatory cytokines, compared with monoinjection of LPS [9]. Further, it has been reported that injection of MDP induces lethal shock in mice challenged with LPS [10]. Furthermore, LPS and MDP synergistically induce proinflammatory cytokine expression in monocyte cell culture [11].

Osteoclast formation is dependent upon stimulation by receptor activator of NF-*κ*B ligand (RANKL) and macrophage colony-stimulating factor (M-CSF) [12]. Additionally, it has been reported that TNF-*α* induces osteoclast formation [13–16] and induces *in vivo* [17, 18]. These cytokines also contribute to LPS-induced osteoclast formation and bone destruction [1, 2, 19–23]; MDP has also been shown to enhance LPS-induced osteoclast formation [24]. These findings suggest that MDP might play an important role in LPS-induced biological activities. Therefore, LPS and MDP are targets of therapies against bacterially induced inflammation.

This review discusses the role of MDP in LPS-mediated biological activities, primarily in relation to osteoclastogenesis.

## 2. Biological Effect of LPS and MDP

LPS localizes within the outer layer of the membrane and is present on the cell surface of Gram-negative bacteria. LPS molecules are made up of three structural components: lipid A, a hydrophobic lipid section, which is responsible for the toxicity of the molecule; a hydrophilic polysaccharide chain that serves as the core of the molecule; and a repeating hydrophilic O-antigenic oligosaccharide side chain that is specific to each bacterial species [25, 26].

LPS induces its action through interactions with toll-like receptor 4 (TLR4) on the cell membrane of a target host cell. First, LPS binds to the serum protein LPS-binding protein (LBP) [27, 28]; then, LPS is transferred to CD14 by the catalytic activity of LBP [29, 30]. Although this LPS-CD14 complex binds to TLR4, the MD-2 molecule is essential for the recognition of LPS by TLR4 [31]. Thus, LPS ultimately binds a CD14/TLR4/MD-2 receptor complex; this is present in many cell types, including monocytes, dendritic cells, macrophages, and B cells. When stimulated by LPS, these cells produce inflammatory cytokines, nitric oxide, and prostaglandin (PGE) [32–36]. Through this mechanism, LPS induces production of many local factors, including TNF-*α* and IL-1, from macrophages and other cells involved in mediating the inflammatory response within tissues [37, 38].

**Figure 1:**
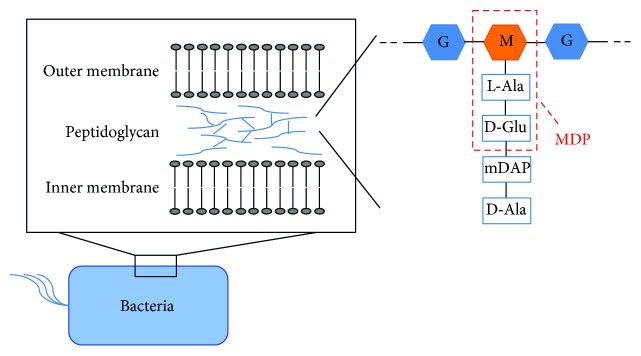
Schematic structure of peptidoglycan and MDP. PGN, a major component of the bacterial cell membrane, is a crystal lattice structure formed by the combination of linear chains of two alternating amino sugars, GlcNAc and MurNAc. MDP consists of MurNAc and two amino acids, l-Ala and d-Glu. M: MurNAc; G: GluNAc.

PGN, another major component of the bacterial cell membrane, is a crystal lattice structure formed by the combination of linear chains of two alternating amino sugars, N-acetylglucosamine (GlcNAc) and N-acetylmuramic acid (MurNAc) [39, 40]. MDP is the minimal essential structural unit responsible for the immunological activity of a wide variety of PGNs (Figure 1). Nucleotide-binding oligomerization domain (NOD) 1 and NOD2 are involved in the recognition of PGN in the cytosol of the cells. MDP is recognized via NOD2 [41, 42]. MDP is also the most basic structure required to maintain the efficacy of Freund's complete adjuvant (FCA). FCA induces both humoral and cellular immune responses. However, the toxicity of FCA is very strong, which makes it difficult to use for clinical applications. Therefore, investigators determined that the smallest biologically active component in FCA was tripeptide-monosaccharide MDP [43] and that this compound maintains adjuvant activity. Thus, MDP replaced FCA in protocols requiring the induction of both humoral and cellular activities. However, MDP cannot induce immunoglobulin production when it is the sole adjuvant [43–45].

When MDP is used as the sole adjuvant, it enhances the expression of cell adhesion molecules and antigen presentation. Therefore, phagocytic activity, antimicrobial activity, and antibody-mediated cytotoxicity are enhanced [46–51]. Additionally, MDP induces immune responses through increased cytokine production, enhancing the differentiation and proliferation of T lymphocytes and subsequent protection against foreign intruders [52–55]. Therefore, MDP serves as an effective adjuvant and may be used to boost the potency of drugs and vaccines.

It has been reported that preexposure to MDP increases immune responses to later challenges. Notably, MDP induced expression of TNF-*α* when injected into mice [9]; in subsequent studies, MDP-induced production of TNF-*α* resulted in lethal shock in mice that were challenged with LPS [10]. In addition, MDP has been shown to synergistically enhance production of proinflammatory cytokines elicited by LPS stimulation of human monocyte cells [11]. Several studies have reported this synergistic effect when MDP is combined with LPS; it was observed in studies of primary cells, such as peripheral blood mononuclear cells (PBMCs), purified monocytes, and various cell lines *in vitro* [11, 56–61].

In other investigations, MDP was reported to enhance the protective response of interferon- (IFN-) *α* and IFN-*β* against encephalomyocarditis virus infection [62]. MDP conjugated to PolyG (a 10-mer polyguanylic acid) enhanced the secretion of IL-6, IL-1, TNF-*α*, and nitric oxide; this resulted in the activation of macrophages with tumoricidal activity [63, 64]. Further, exposure to paclitaxel-conjugated MDP increased antitumor activity [65] and enhanced the expression of TNF-*α* and IL-12 by mouse peritoneal macrophages [66].

MDP and its derivatives, such as murabutide (MB), have potential for a variety of clinical applications. MB enhances resistance against bacterial and viral infections, such as infection by human immunodeficiency virus (HIV) [67–72]. MB stimulation inhibited HIV replication in macrophages via NOD2 signaling [73]. Human PBMCs that were stimulated *in vitro* with IL-2 and murabutide showed synergistic induction of IFN-*γ* expression [74]. Combined administration of MB and IL-2 into Meth-A sarcoma-bearing mice resulted in significant tumor inhibition and complete tumor regression in 70% of treated mice [74].

## 3. The Role of MDP and LPS in Osteoclast Formation and Bone Remodeling

Osteoclasts develop from myeloid lineage cells; they function to resorb bone and control bone remodeling [12]. In osteolytic diseases, both the formation and activity of osteoclasts are exceptionally stimulated [75]. The osteoclast is considered central to diseases involving bone erosion, such as rheumatoid arthritis [75], periprosthetic bone loss [76], postmenopausal osteoporosis [77, 78], and periodontal disease [1, 79].

LPS induces production of proinflammatory cytokines, such as TNF-*α* and IL-1, from macrophages and other cells in affected tissues [7]. The production of TNF-*α* and IL-1 is associated with LPS-induced osteoclast formation and bone destruction *in vivo* and *in vitro* [1, 2, 19–22]. Further, LPS stimulates osteoblasts to produce RANKL [80].

In contrast, a variety of cytokines, including IL-4, IL-10, IL-12, IL-13, IL-18, and IFN-*γ*, are able to inhibit osteoclast formation [81]. Furthermore, some cytokines, such as IL-4, have direct inhibitory effects on osteoclast formation by modifying the effects of RANKL and TNF-*α* on osteoclast precursor cells [82–85].

Several papers have reported that LPS-induced osteoclast formation *in vivo* is inhibited by exposure to a variety of cytokines, such as IL-4, IFN-*γ*, IL-12, and IL-37. IL-4 and IFN-*γ* were found to directly inhibit LPS-induced differentiation of osteoclast precursors into osteoclasts [86]. *In vivo* IL-12 stimulation inhibits LPS-induced osteoclastogenesis. mRNA levels of both Fas and FasL increased in mice that were coadministered LPS and IL-12; this might lead to apoptotic changes in osteoclastogenesis-related cells through Fas/FasL interactions [87]. *In vivo* IL-37 stimulation inhibited LPS-induced osteoclast formation and bone resorption via inhibition of LPS-induced osteoclast-related cytokines. However, IL-37 might act indirectly to inhibit osteoclast formation by osteoclast precursor cells and RANKL expression by stromal cells [88].

MDP stimulation can enhance osteoclast formation that is initially induced by LPS, IL-1*α*, or TNF-*α*, but not by 1*α*,25-dihydroxy-vitamin-D3 (1*α*,25(OH)2D3) or PGE2. Furthermore, MDP upregulated RANKL expression in osteoblasts treated with LPS or TNF-*α*, but not with 1*α*,25(OH)2D3 [89]. However, MDP alone cannot induce osteoclast formation in cocultures of primary murine osteoblasts and hematopoietic cells.

The *in vitro* effects of PGN on LPS-induced osteoclast formation and bone resorption have been investigated. Furthermore, during a set of *in vivo* studies, PGN significantly induced osteoclast formation and bone resorption in mice coinjected with LPS [90]. Since MDP is the minimal essential structural unit responsible for the immunological activity of PGN, we suspect that MDP plays an important role in the ability of PGN to enhance LPS-induced osteoclast formation and bone resorption.

Recently, the effect of MDP in LPS-induced osteoclast formation and bone resorption has been reported. In that study, LPS was administered as a monoinjection, or as a coinjection with MDP, into the supracalvariae of mice. Compared with mice that received a monoinjection of LPS, mice that received a coinjection of LPS and MDP exhibited an increase in the following parameters: number of osteoclasts, levels of cathepsin K mRNA and tartrate-resistant acid phosphatase (TRAP) mRNA, ratio of bone destruction area and levels of TRAP 5b (TRACP5b), and C-terminal telopeptide fragments of type I collagen (a marker of bone resorption). In contrast, exposure to MDP alone had no effect on osteoclastogenesis in PTH-stimulated mice. These results suggest that MDP enhances LPS-induced osteoclast formation and bone resorption [24].

LPS has been shown to enhance the production and secretion of RANKL by osteoblasts [80]. A later *in vitro* study of osteoblasts that were cultured with LPS alone, or with a combination of LPS and MDP, indicated that MDP stimulation enhances LPS-induced expression of RANKL mRNA in osteoblasts [89]. Another study showed that MDP enhances LPS-induced expression of RANKL mRNA in stromal cells [24], which supported previous findings. However, these results indicate that MDP alone cannot induce RANKL expression, either *in vitro* or *in vivo*, and that MDP is only able to enhance the effects of LPS exposure (e.g., RANKL expression). In contrast, MDP cannot enhance PTH-induced osteoclast formation and bone resorption, suggesting that MDP affects LPS signaling but not PTH-induced signaling [24].

LPS is recognized by TLR4 on the host cell surface [91–93]. TLR4 signaling generates proinflammatory host defense processes [94–97]. During osteoclast formation, stromal cells, such as osteoblasts, express RANKL. It has been reported that LPS induces an increase in TLR4 expression in stromal cells. Furthermore, MDP has been shown to enhance LPS-induced upregulation of TLR4 expression in stromal cells. However, PTH stimulation does not enhance TLR4 expression, suggesting that MDP enhances LPS signaling by increasing TLR4 expression [24]. Taken together, these results indicate that MDP enhances LPS-induced RANKL expression and TLR4 expression in stromal cells.

Costimulation of osteoblasts, using a combination of NOD1 or NOD2 ligands and TLR2 or TLR4 ligands, has been reported to enhance the expression of cyclooxygenase- (COX-) 2, PGE2, IL-1*β*, IL-6, and IL-8 through an increase in TRAF6 expression [98]. MDP stimulation synergistically increased RANKL expression in osteoblasts that were costimulated with LPS, IL-1*α*, and TNF-*α*; this costimulation enhances osteoclast formation [89]. LPS has been reported to induce the phosphorylation of MAPKs (ERK, P38, and JNK) in osteoblasts [99, 100]. MDP has also been reported to enhance LPS-stimulated ERK1/2 phosphorylation in osteoblasts [89]. Furthermore, although MDP alone cannot activate MAPKs, MDP enhances LPS-induced MAPK phosphorylation in stromal cells. Thus, MDP appears to enhance LPS-related signal transduction.

**Figure 2:**
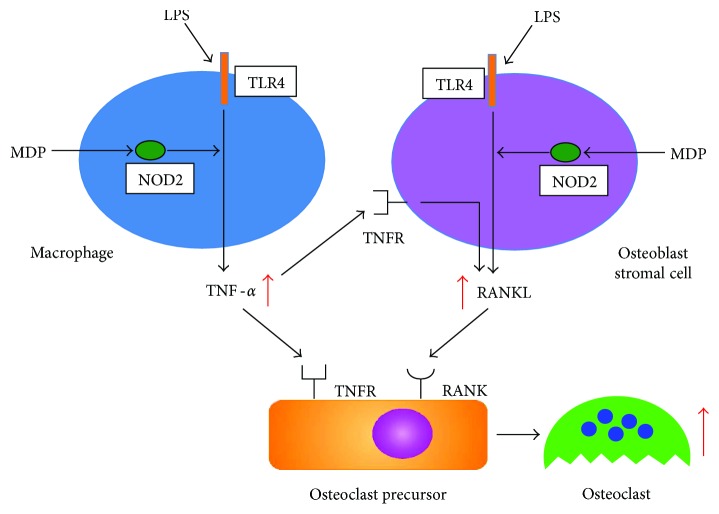
Schematic of the role of MDP in LPS-induced osteoclast formation. LPS induces TNF-*α* expression; MDP further enhances this TNF-*α* expression. MDP-enhanced, LPS-induced TNF-*α* may lead to an increase in RANKL expression by stromal cells. Furthermore, LPS also acts to induce RANKL expression; MDP further enhances this RANKL expression in stromal cells. The MDP-enhanced, LPS-induced TNF-*α* production synergistically interacts with MDP-enhanced, LPS-induced RANKL, thereby leading to increased induction of osteoclastogenesis. Therefore, the role of MDP in LPS-induced osteoclast formation *in vivo* may be to strongly promote the induction of this process.

LPS exposure induces TNF-*α* expression in macrophages and other immune cells; MDP exposure enhances this LPS-induced TNF-*α* expression. TNF-*α* stimulation increases RANKL expression by stromal cells [18, 101–103]. Therefore, LPS-induced TNF-*α*, which has been further enhanced by MDP, may greatly increase RANKL expression by stromal cells. Furthermore, LPS acts to induce RANKL expression in stromal cells, and MDP also enhances this LPS-induced RANKL expression. TNF-*α* promotes osteoclast formation by osteoclast precursors by synergizing with RANKL [104, 105] at the signal transduction level [15, 18, 104–106]. This suggests that TNF-*α* synergistically increases RANKL-induced osteoclast formation. Therefore, LPS-induced TNF-*α* production is enhanced by MDP and synergistically interacts with RANKL, leading to a large induction of osteoclastogenesis (Figure 2). Thus, the role of MDP in LPS-induced osteoclast formation *in vivo* may be to strongly promote the induction of this process.

## 4. Conclusions

LPS plays a key role in the induction of important biological activities such as inflammation. LPS induces osteoclast formation during inflammatory processes, such as periodontal disease. MDP exposure enhances LPS-induced biological activities, including osteoclast formation and bone destruction, through a mechanism that is gradually becoming clear. Therefore, targeted therapies against LPS and MDP could serve an important role in management of inflammatory disease processes. Further studies are required to fully understand the mechanisms of LPS- and MDP-mediated biological activities, especially osteoclast formation.
